# Concurrent Effects of Drought and Heat Stresses on Physio-Chemical Attributes, Antioxidant Status and Kernel Quality Traits in Maize (*Zea mays L.*) Hybrids

**DOI:** 10.3389/fpls.2022.898823

**Published:** 2022-05-11

**Authors:** Muhammad Irfan Yousaf, Muhammad Waheed Riaz, Yurong Jiang, Muhammad Yasir, Muhammad Zahid Aslam, Sabir Hussain, Syed Awais Sajid Shah, Aamar Shehzad, Gulfam Riasat, Muhammad Aamir Manzoor, Imran Akhtar

**Affiliations:** ^1^Cotton Research Station (CRS), Bahawalpur, Pakistan; ^2^Maize and Millets Research Institute (MMRI), Sahiwal, Pakistan; ^3^State Key Laboratory of Subtropical Silviculture, Zhejiang A&F University, Hangzhou, China; ^4^Zhejiang Provincial Key Laboratory of Resources Protection and Innovation of Traditional Chinese Medicine, Zhejiang A&F University, Hangzhou, China; ^5^College of Advanced Agricultural Sciences, Zhejiang A&F University, Hangzhou, China; ^6^Maize Research Station, Ayub Agricultural Research Institute, Faisalabad, Pakistan; ^7^School of Life Sciences, Anhui Agricultural University, Hefei, China; ^8^Regional Agricultural Research Institute, Bahawalpur, Pakistan

**Keywords:** heat stress, kernel quality, water-deficient, antioxidant activity, maize, maize hybrids, stress physiology

## Abstract

Maize is one of the most important field crops considering its utilization as food, feed, fodder, and biofuel. However, the sustainability of its production is under serious threat of heat and drought stresses, as these stresses could hamper crop growth, causing a significant loss to kernel yield. The research study was carried out at Maize and Millets Research Institute, Yusafwala-Sahiwal for two consecutive spring seasons (2019–20 and 2020–21) under a split-split plot design. The current study explained the individual and combined effects of drought and heat stresses on morphology, phenology, physiology, reactive oxygen species (stocktickerROS), antioxidant status, and kernel quality traits in four indigenous (YH-5482, YH-5427, YH-5404, and YH-1898) and one multinational maize hybrid (P-1543). Stress treatments, i.e., drought, heat, and drought+heat, were applied ten days before tasseling and lasted for 21 days. The results revealed the incidence of oxidative stress due to overproduction of Hydrogen peroxide; H_2_O_2_ (control: 1.9, heat+drought: 5.8), and Malondialdehyde; stocktickerMDA (control: 116.5, heat+drought: 193), leading to reduced photosynthetic ability (control: 31.8, heat:16.5), alterations in plant morphology, decrease in kernel yield (control: 10865 kg ha^–1^, heat+drought: 5564 kg ha^–1^), and quality-related traits. Although all the stress treatments induced the accumulation of stress-responsive osmolytes and enzymatic antioxidants to cope with the negative impact of osmotic stress, the effect of combined drought + heat stress was much higher. The overall performance of indigenous maize hybrid YH-5427 was much more promising than the other hybrids, attributed to its better tolerance of drought and heat stresses. Such stress tolerance was attributed to maintaining photosynthetic activity, a potent antioxidant and osmolyte-based defense mechanisms, and minimum reductions in yield-related traits, which assured the maximum kernel yield under all stress treatments.

## Introduction

Changing climatic conditions impose a severe threat to crops’ growth and productivity by changing their growth pattern and ability to withstand harsh climatic conditions (Calleja-Cabrera et al., 2020). The continuous decline in cultivated areas and productivity exacerbated the effects of climate change. Among these, drought and heat stress are the two most critical abiotic stresses that determine the overall crop productivity and, ultimately, the region’s food security. These abiotic stresses are one of the major causes of the reduction in productivity of all major cereal crops worldwide, accounting for 60% of the world’s energy supply (Zandalinas et al., 2018). Moreover, about 31% to 81% reduction in grain yield is reported to be directly linked to heat and drought stresses in major staple crops around the globe (Farooq et al., 2017). Recent studies revealed that the combined effects of drought + heat stress are more radical in reducing overall crop productivity than individual stresses in maize crop (Meseka et al., 2018; Hussain et al., 2019; Nelimor et al., 2020). Although combined effects of drought and heat stresses are more severe in comparison to their individual stresses, however, the individual heat and drought stresses also caused significant yield reduction in different field crops including maize (Riaz et al., 2021). This substantial decrease in grain yield under drought and heat stress conditions is associated with the decrease in their photosynthetic ability, membrane stability, leaf area, plant biomass, heat and water use efficiency, and imbalance between source-sink ratio (Barnabás et al., 2008; Lipiec et al., 2013). However, the actual impact of these stresses is associated with the intensity, duration, species, and stage of plant exposure to stress (Fahad et al., 2017).

Heat stress alone or in combination with water-deficient conditions increases the temperature of plant tissues and hampers the functioning of crucial physiological processes, including photosynthesis and respiration. Several studies unveiled that heat stress accompanies water deficit stress due to increased water losses from the soil and plant surfaces to meet evapotranspiration demands (Pei et al., 1998; Wahid et al., 2007). In the case of the wheat crop, exposure to heat stress at the reproductive stage hinders crop growth and development by reducing photosynthetic efficiency, leaf water contents, and thousand-grain weight, which ultimately reduces the grain yield (Riaz et al., 2021). Recently, Fahad et al. (2017) and Hussain et al. (2019) concluded that both drought and heat stresses trigger similar physiological responses in maize plants; however, the worse effects of their combined action suggest the existence of a conserved defense mechanism between different plant species to cope with individual drought and heat stresses. Maize, similar to other crops, possesses several defense mechanisms to counter the negative impacts of abiotic stresses, including activation of stress-responsive genes and production of stress protein (Heat shock protein), accumulation of compatible osmolytes (proline, sugar, etc.), activation of enzymatic, i.e., total superoxide dismutase (T-SOD), Catalase (CAT) and Peroxidase (POD) and non-enzymatic antioxidants, etc. (Hussain et al., 2019). However, the efficiency of defense mechanisms varies from species to species and also genotype to genotype.

Maize is one of the most essential and productive cereal crops. It is also called the 4F crop due to its utilization as food, feed, fodder, and fuel. Considering its consumption, 65% of maize production is used in the poultry and livestock industry, 35% in wet and dry milling, and human food. In Pakistan, maize is grown in two seasons; Spring and Autumn. About 85% of the production comes from the Province of Punjab. In 2020-21, maize was sown on an area of 1.418 million hectares and 8.465 million tons of production was obtained with an average of 5970 kg ha**^–^**^1^ (ESP, 2021). Although maize originated from the tropics, it is sensitive to drought and heat stress conditions, especially late vegetative and reproductive stages. Pakistan is one of those countries which are severely hit by climate change effects, especially water scarcity and high temperature (ADB, 2017). It is projected that drought and heat stresses could decrease maize yield as low as 87% and 65%, respectively (Kamara et al., 2003; Lobell et al., 2011). Improper irrigation water scheduling could also be an important factor along with drought stress in reducing the maize yield ranging from 30 to 90% (Çakir, 2004; Amiri et al., 2015).

In several studies, the impact of individual heat and drought stresses was quantified based on their morpho-physiological parameters, however, the combined effects of drought and heat stresses are poorly studied for kernel yield and quality-related traits, especially for the heat and drought-prone regions of the world like Pakistan. Moreover, most of the previous studies were based on crop prediction models, not the actual field studies. The current field study was designed to elucidate the actual mechanism involved in individual and combined drought and heat stress tolerance. The specific objectives of this study were (1) to evaluate the effects of individual drought and heat stresses as well as their cumulative impact on morphology, phenology, physiology, kernel quality, and biochemistry in maize hybrids (2) to investigate the basis of heat and drought tolerance in maize (3) to evaluate the comparative performance of five maize hybrids under individual drought and heat stresses along with their interactive effects.

## Materials and Methods

### Plant Materials and Growing Conditions

The experiment was conducted at Maize and Millets Research Institute, Yusafwala- Sahiwal, Pakistan (longitude 73°12′39.3″E, latitude 30°40′57.1″E, altitude 173 meters) under field conditions for two consecutive spring seasons (Spring 2019-20 and 2020-21). Seeds of four single cross maize hybrids (YH-5482, YH-5427, YH-5404, and YH-1898) were obtained from Maize Hybrid Development Group, Maize and Millets Research Institute, Yusafwala, while one single cross maize hybrid (P-1543) was obtained from Corteva Seed (Pvt. Limited). The soil, used for the study, was clay loamy, with a pH of 7.9–8.0 and a good drainage capacity.

### Stress Treatments

Maize hybrids were sown under four field conditions. (a) control/optimal treatment; sowing was done in mid-February in both spring seasons, (b) heat stress treatment; heat stress was applied by delaying the sowing up to late-March so that reproductive phase of maize crop may experience high-temperature stress 40°C ≤, (c) drought treatment; drought stress was imposed by withholding two consecutive irrigation (re-irrigation after 21 days) at flowering stage, (d) combined drought + heat stress treatment; for this stress, sowing was done in the third week of March so that high-temperature coincide with the reproductive phase of maize crop and at the same time, two consecutive irrigations were skipped to develop water stress as well. These treatments were arranged in a randomized complete block design (RCBD) with three replications under a split-split plot arrangement.

### Sample Collection for Biochemical Analysis

Fully extended and healthy leaves, fourth from the top, from ten random plants were collected from each replication three weeks after the imposition of stress. Leaves were then washed with distilled and froze under liquid nitrogen (N_2_) and stored at −80^°^C for the measurement of different biochemical compounds.

### Measurement of Yield-Related Morpho-Agronomical Traits

For morpho-physiological traits, ten healthy, mature, random, and guarded plants were selected for the collection of data. Days to 50% anthesis (DA) and silking (DS) were recorded by counting the days from sowing to the completion of 50% anthesis and silking of maize hybrids, respectively. Plant height (PH), thousand kernel weight (TKW), and the number of kernels per cob (NK/C) were recorded using standard procedures. Kernel yield is the ultimate end product and is measured according to the given formula; (Tandzi and Mutengwa, 2020)


$$\mathrm{Grain}\,\mathrm{Yield}\,\left(\mathrm{Kg}\,{\mathrm{ha}}^{-1}\right)$$

$$=\frac{\mathrm{Fresh}\,\mathrm{ear}\,\mathrm{weight}\,\left(\mathrm{Kg}\,{\text{plot}}^{-1}\right)\times \left(100-\mathrm{MC}\right)\times 0.8}{\left(100-15\right)\times A\,r\,e\,a\,H\,a\,r\,v\,e\,s\,t\,e\,d/p\,l\,o\,t}\times 10000$$

where;

MC = Moisture contents of kernels.

### Measurements of Physiological Parameters

Photosynthetic components were recorded from the intact ear leaves after 15 days of impositions of stress treatments. Net photosynthetic rate (Pn), Stomatal conductance (Ci), and transpiration rate (Tr) were measured through an Infra-red gas analyzer (IRGA) using a CI-320 handheld photosynthesis system (CID Bio-Science Inc., Washington State, United States) as suggested and used by Yousaf et al. (2021). The concentrations of chlorophyll a (Chl a), chlorophyll b (Chl b), and total carotenoid contents (Carotenoids) were measured in (mg g**^–^**^1^ dry weight) through spectroscopy. These photosynthetic pigments were extracted from leaf samples in 100% acetone. The absorbance of these leaf extracts at 470, 644.8, and 661.6 nm was measured using Shimadzu UV-1280 UV-VIS spectrophotometer, and values were calculated using adjusted extinction coefficients (Lichtenthaler, 1987; Peng and Liu, 1992).

### Measurements of Biochemical Parameters

#### Proline Contents

Proline contents were determined from the leaves of controlled and stress-treated plants by following the acid ninhydrin method suggested by Efeoglu et al. (2009). Proline was extracted from the leaf samples according to the method suggested by Hussain et al. (2019), and obtained values were compared with the known amount of proline.

#### Malondialdehyde and H_2_O_2_ Accumulation

Malondialdehyde (MDA), a widely used marker of oxidative stress injury to lipids, was measured through the thiobarbituric (TBA) method according to Heath and Packer (1968). Leaf samples were ground in 2 cm^3^ of 0.1% trichloroacetic acid and centrifuged at 1,000 × g for 10 min and 1 cm^3^ from the supernatant was mixed with 4 cm^3^ solution having 0.5% (w/v) thiobarbituric acid and 20% (w/v) trichloroacetic acid. The mixture was then heated over boiling water, rapidly cooled over ice, and centrifuged for 15 min at 1000 × *g*. The absorbance of the obtained sample was then measured at 532 and 600 nm. MDA concentration was calculated after subtracting nonspecific absorbance at 600 nm using an extinction coefficient of 155 mM^–1^cm^–1^. MDA contents were represented as nmol g**^–^**^1^ (Fresh weight). Leaf samples (0.5 g) were homogenized in 5 mL of 5% trichloroacetic acid (TCA), filtered, and centrifuged at 12000 × *g* for 15 min. The supernatant was used for the determination of H_2_O_2_. The H_2_O_2_ contents were determined according to the procedure developed by Sergiev et al. (1997). The reaction mixture consisting of leaf extract, 2.5 mM potassium-phosphate buffer (pH 7.0) and 0.5 M potassium iodide (KI). After keeping the mixture in the dark for 1 h, the amount of H_2_O_2_ was determined through a spectrophotometer at 415 nm by reference to a standard curve prepared with H_2_O_2_ solutions.

#### Enzymatic Antioxidant Determination (Catalysis and Total Superoxide Dismutase)

Leaf samples (0.5 g) from ten random plants were ground meticulously in 10 ml of 50 mm phosphate buffer (pH 7.8). The samples were centrifuged at 15000 ×*g* for 10 min at 4^°^C. The supernatant was used for the determination of antioxidant activities. The Catalase activity (CAT) was measured by the method given by Chance and Maehly (1955) with a minor modification. The reaction solution (3 ml) consisted of 50 mM phosphate buffer (pH 7.8), 59 mM H_2_O_2_, and 0.1 ml sample extract. After the reaction initiation by adding enzyme extract, its absorbance was read at 240 nm with an interval of 20 s for 2 min. The activity of each enzyme was expressed on a protein weight basis.

#### Kernel Quality Parameters

Kernel quality parameters, i.e., kernel protein content percentage, kernel oil content percentage, and kernel starch content percentage, were measured through Near-Infrared Spectroscopy (NIR) using Inframatic 9200, Partin instruments, Sweden. An average of three samples (750g each) from each replication was used to estimate the contents of kernel quality parameters. The parameters were expressed in percentages (%).

### Statistical Data Analysis

The collected data for kernel yield and its associated traits were statistically analyzed for combined analysis of variance (ANOVA) and correlation coefficient through Statistix 8.1 and XLSTAT statistical package (Steel and Torrie, 1997), whereas statistical differences between control and stress treatments were calculated through Duncan’s multiple range test. Biplot analysis was used to characterize maize hybrids under different treatments by using XLSTAT 2020. Furthermore, Microsoft Excel 2019 was used for the graphical representation of data.

## Results

### Metrological Data

**FIGURE 1:**
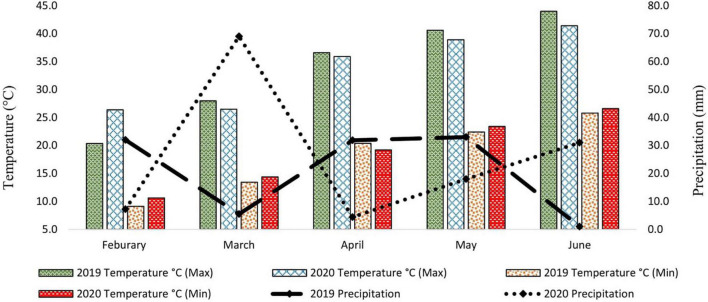
Metrological Data of two spring growing seasons (2019–20 and 2020–21).

**FIGURE 2:**
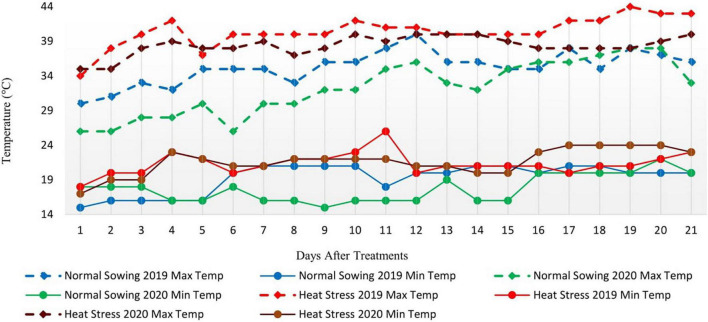
Minimum and Maximum Temperature during Normal and heat stress Conditions (2019 and 2020).

Metrological data were recorded daily for two consecutive whole growing seasons. Data for average daily maximum temperature (°C), average daily minimum temperature (°C), and precipitation (mm) were recorded and presented monthly (Figure 1). The data showed that the average daily maximum temperature was highest in May and June when the crop was at the reproductive phase due to late sowing (late sowing was used as a proxy for heat stress). Furthermore, the average daily maximum temperature in heat stress treatment (late sowing) was significantly higher than optimal sowing in both seasons (Figure 2). Similarly, the average daily minimum temperature was also higher for stress treatment than for optimal sowing.

### Kernel Yield and Associated Morpho-Agronomical Traits

**FIGURE 3:**
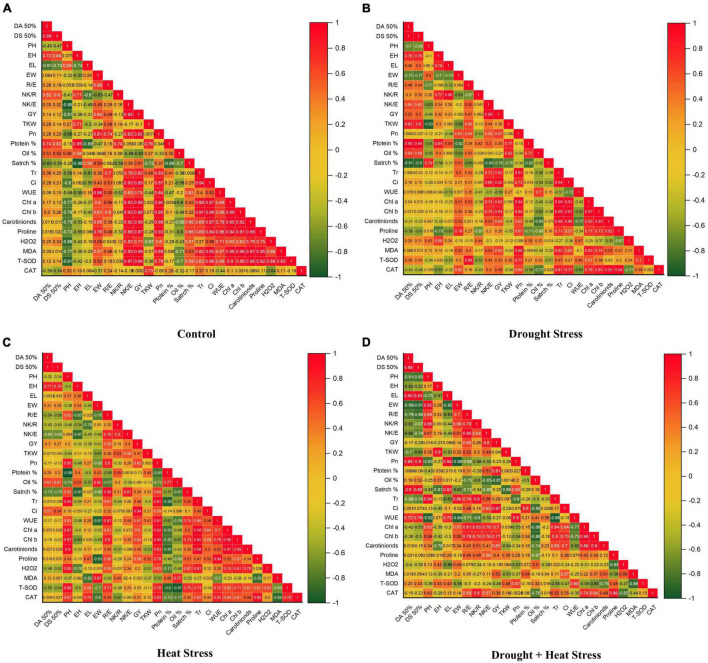
Correlation coefficient graphs of morpho-physiological, biochemical, and stress-related parameters in five maize hybrids at the pre-anthesis stage under **(A)** control **(B)** drought stress **(C)** heat Stress **(D)** heat + drought stress.

Heat and drought stress conditions alone or in combination restrained the overall growth causing significant variations and reduction in kernel yield and its associated traits (Table 1). Compared to control, combined heat + drought stress significantly reduced all the studied traits, i.e., days to 50% anthesis, days to 50% silking, plant height, ear height, ear width, number of rows per ear, number of kernels per row, number of kernels per ear, thousand kernel weight and ultimately kernel yield (Table 2). At individual stress levels, drought stress proved more critical for days to 50% anthesis, days to 50% silking, plant height, ear height, number of kernels per ear, thousand kernel weight, and kernel yield than heat stress (Table 2). However, ear length and width were more affected by heat stress than drought. However, the number of rows per ear and the number of kernels per row showed mixed/unstable behavior for both stresses. Correlation coefficient graphs revealed the presence of a significant positive correlation of grain yield with the days to 50% anthesis, days to 50% silking, and the number of kernels per ear under individual heat and drought stress conditions; however, the correlation of days to 50% anthesis and silking with grain yield under combined drought + heat stress was significantly negative along with plant height, ear height and ear length (Figures 3A–D). Under all the stress levels, the agro-morphological performance of two maize hybrids, YH-5427 and YH-5482, was higher than YH-5404, P-1543, and YH-1898, respectively.

**TABLE 1:** Mean Squares (MS) of plant traits in five maize hybrids under individual and combined heat and drought stresses.

Traits	Replication (R)	Years (Y)	Error (R × Y)	Treatments (T)	Error (Y × R × T)	Hybrids (H)	Interaction (T × H)	Error (R × Y × T × H)
**Degree of freedom**	***2***	***1***	***2***	***3***	***12***	***4***	***12***	***64***
Days to 50% anthesis	0.05	10.80	6.6	650.5**	0.11	26.31**	9.81**	0.26
Days to 50% silking	0.49	4.8	3.13	642.7**	0.13	26.81**	9.90**	0.29
Plant height	44.9	28.7	18	5225.2**	18	934.2**	116.0**	6.6
Ear height	1.52	16.3	10.33	895.5**	1.33	945.57**	88.31**	1.3
Ear length	0.63	1.2	1.04	26.2**	0.04	4.94**	1.92**	0.08
Ear width	2.94	2.7	1.74	241.7**	0.74	32.76**	6.06**	0.43
Rows per ear	0.5	0.206	0.14	13.7**	0.14	2.85**	1.12**	0.08
Kernels per row	2.5	1.20	0.8	80.4**	0.2	25.3**	11.8**	0.4
Kernels per ear	568.3	940.8	563.6	65894.7**	71.6	3809.9**	2812.4**	101.1
Thousand kernel weight	209.1	152.3	98.7	21875.0**	9.7	19581.9**	1475.5**	17
Net photosynthetic rate	0.55	0.30	0.25	263.3**	0.05	25.14**	7.96**	0.21
Protein %	0.21	0.300	0.21	5.5**	0.01	0.13**	0.28**	0.05
Oil %	0.029	0.1613	0.095	0.49**	0.005	0.601**	0.081**	0.006
Starch %	1.15	3.19	2.39	40.3**	0.39	68.96**	8.26**	0.54
Transpiration rate	0.006	0.0822	0.064	4.31**	0.004	0.261**	0.108**	0.004
Stomatal conductance	76.9	67.5	58.6	47851.6**	31.6	8254.9**	413.6**	14.5
Water use efficiency	0.2	0.2689	0.12	38.8**	0.02	1.80**	1.32**	0.02
Chlorophyll a	0.021	0.076	0.062	1.24**	0.002	0.114**	0.047**	0.003
Chlorophyll b	0.002	0.03	0.051	0.28**	0.001	0.029**	0.015**	0.001
Carotenoids contents	0.005	0.116	0.091	0.39**	0.001	0.066**	0.014**	0.002
Proline contents	0.36	0.93	0.6	870.7**	0.03	3.41**	2.52**	0.05
H_2_O_2_ contents	0.014	2.7*	0.9	21.05**	0.009	0.005*	0.13	0.003**
MDA contents	78.4	18.5*	7.7	7314.1**	7.7	40.8	72.7	6.3
Total SOD (T-SOD)	9.35	18.5	9.49	2970.2**	0.49	47.93**	15.41**	0.85
Catalysis (CAT)	0.1	2.7	0.91	0.83*	0.31	2.7	0.01	0.02
Kernel yield (kg ha**^–^**^1^)	80502	5494.5	4613	2836465**	444652	5494.53	4613**	1286

*Only selected sources of variations are given in the table, otherwise the table fitness had to be compromised. Moreover, the ANOVA table is only to show the differences between different sources of variations.*

*Significant changes are highlighted by an asterisk (*); *P ≤ 0.05, **P ≤ 0.01; ns: non-significant.

**TABLE 2:** Effects of individual and combined heat and drought stresses on morpho-physiological and stress-related parameters in maize hybrids.

Hybrids	Treatment	DA	DS	PH	EH	EL	EW	R/E	NK/R	NK/E	GY	TKW
YH-5482	*Control*	74.1 ± 0.219	77.1 ± 0.228	178.3 ± 1.083	96.5 ± 0.747	18.9 ± 0.115	49.9 ± 0.420	17.5 ± 0.184	39.9 ± 0.260	699.4 ± 3.812	10687 ± 55.456	288.8 ± 4.076
	*Drought*	70.8 ± 0.213	73.6 ± 0.264	184.7 ± 1.970	94.2 ± 0.413	19.0 ± 0.118	47.0 ± 0.390	17.5 ± 0.259	39.7 ± 0.649	695.9 ± 5.902	9071 ± 36.410	343.2 ± 0.734
	*Heat*	71.8 ± 0.198	74.6 ± 0.173	211.7 ± 1.146	104.5 ± 0.523	19.9 ± 0.338	47.3 ± 0.577	18.0 ± 0.135	39.1 ± 0.257	606.7 ± 6.989	8418 ± 75.459	361.4 ± 2.999
	*Heat + Drought*	72.8 ± 0.199	75.7 ± 0.176	195.0 ± 2.424	113.0 ± 1.300	18.7 ± 0.151	42.9 ± 0.604	15.8 ± 0.048	41.1 ± 0.199	629.5 ± 4.833	7869 ± 19.440	329.4 ± 1.891
YH-5427	*Control*	72.2 ± 0.419	75.3 ± 0.390	192.3 ± 0.641	92.0 ± 0.805	18.3 ± 0.061	49.6 ± 0.165	16.2 ± 0.380	35.6 ± 0.407	643.7 ± 7.653	10865 ± 96.616	282.5 ± 1.631
	*Drought*	72.8 ± 0.234	76.0 ± 0.267	225.0 ± 2.151	90.0 ± 0.570	18.0 ± 0.177	46.8 ± 0.153	15.5 ± 0.162	38.3 ± 0.196	620.5 ± 3.177	9278 ± 65.711	341.2 ± 0.808
	*Heat*	74.5 ± 0.336	77.7 ± 0.336	201.8 ± 1.166	80.5 ± 0.374	20.6 ± 0.321	47.7 ± 0.812	16.8 ± 0.097	38.0 ± 0.381	662.1 ± 14.581	8795 ± 35.245	367.0 ± 2.367
	*Heat + Drought*	75.7 ± 0.001	78.7 ± 0.111	215.5 ± 1.761	98.5 ± 0.350	20.2 ± 0.149	50.1 ± 0.595	16.2 ± 0.203	35.1 ± 0.368	573.8 ± 1.631	8345 ± 18.200	367.9 ± 0.724
YH-5404	*Control*	72.8 ± 0.243	76.0 ± 0.271	225.0 ± 1.984	90.0 ± 0.900	18.0 ± 0.265	46.8 ± 0.156	15.5 ± 0.089	38.3 ± 0.334	620.5 ± 11.070	9665 ± 174.715	341.2 ± 1.970
	*Drought*	74.5 ± 0.396	77.7 ± 0.306	201.8 ± 1.197	80.5 ± 0.476	20.6 ± 0.116	47.7 ± 0.491	16.8 ± 0.099	38.0 ± 0.232	662.1 ± 7.837	7687 ± 22.547	367.0 ± 2.711
	*Heat*	75.7 ± 0.202	78.7 ± 0.202	215.5 ± 2.205	98.5 ± 0.494	20.2 ± 0.100	50.1 ± 0.160	16.2 ± 0.092	35.1 ± 0.853	573.8 ± 3.362	7297 ± 39.974	367.9 ± 3.975
	*Heat + Drought*	69.2 ± 0.341	72.3 ± 0.545	163.9 ± 2.047	71.2 ± 1.160	17.8 ± 0.000	44.0 ± 0.617	14.5 ± 0.206	36.9 ± 0.202	516.3 ± 2.885	5624 ± 61.860	220.4 ± 4.953
YH-1898	*Control*	74.5 ± 0.428	77.7 ± 0.487	201.8 ± 3.082	80.5 ± 0.465	20.6 ± 0.235	47.7 ± 0.578	16.8 ± 0.397	38.0 ± 0.207	662.1 ± 3.842	9503 ± 87.160	367.0 ± 4.893
	*Drought*	75.7 ± 0.235	78.7 ± 0.227	215.5 ± 1.127	98.5 ± 0.408	20.2 ± 0.273	50.1 ± 0.393	16.2 ± 0.131	35.1 ± 0.418	573.8 ± 6.090	7892 ± 41.459	367.9 ± 2.898
	*Heat*	69.2 ± 0.203	72.3 ± 0.203	163.9 ± 2.229	71.2 ± 0.386	17.8 ± 0.131	44.0 ± 0.315	14.5 ± 0.157	36.9 ± 0.315	516.3 ± 8.653	7943 ± 95.828	220.4 ± 3.143
	*Heat + Drought*	70.1 ± 0.338	73.2 ± 0.338	161.4 ± 1.931	84.6 ± 0.708	17.7 ± 0.208	45.6 ± 0.242	14.2 ± 0.049	33.9 ± 0.264	481.3 ± 3.966	6271 ± 21.434	242.4 ± 0.949
P-1543	*Control*	75.7 ± 0.251	78.7 ± 0.251	215.5 ± 2.488	98.5 ± 0.574	20.2 ± 0.117	50.1 ± 0.596	16.2 ± 0.215	35.1 ± 0.468	573.8 ± 3.826	9876 ± 158.90	367.9 ± 1.226
	*Drought*	69.2 ± 0.235	72.3 ± 0.220	163.9 ± 0.577	71.2 ± 0.780	17.8 ± 0.168	44.0 ± 0.000	14.5 ± 0.168	36.9 ± 0.274	516.3 ± 1.572	7234 ± 80.048	220.4 ± 3.684
	*Heat*	70.1 ± 0.212	73.2 ± 0.184	161.4 ± 1.180	84.6 ± 0.317	17.7 ± 0.126	45.6 ± 0.331	14.2 ± 0.169	33.9 ± 0.281	481.3 ± 4.988	6371 ± 84.725	242.4 ± 3.886
	*Heat + Drought*	68.3 ± 0.200	71.7 ± 0.467	179.6 ± 2.213	81.6 ± 0.291	17.4 ± 0.182	42.9 ± 0.384	14.8 ± 0.167	34.5 ± 0.114	510.1 ± 2.733	5564 ± 53.391	310.7 ± 2.579

*DA: Days to anthesis; DS: Days to silking; PH: Plant height (cm); EH: Ear height (cm); EL: Ear length (cm); EW: Ear width (mm); R/E: Number of rows per ear; NK/R: Number of kernels per row; NK/E: Number of kernels per ear; GY: Grain yield (Kg ha^–1^); TKW: Thousand kernel weight (g). The values are obtained by pooling the mean data of three replicates of two spring growing seasons (2019–20 and 2020–21). ±SE (Standard Error).*

### Physiological Traits

**FIGURE 4:**
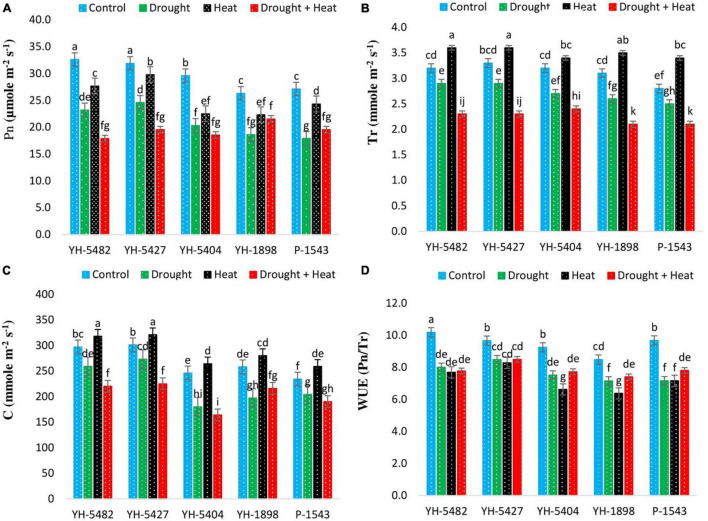
Effect of heat, drought, and heat +drought stresses on **(A)** Net Photosynthetic rate **(B)** Transpirational rate **(C)** Stomatal Conductance, and **(D)** Water use efficiency in five maize hybrids. Capped bars above means represent the ± SE of three replicates.

The response of different plant physiological parameters to drought and heat stresses was quite alarming in almost all maize hybrids. Results revealed that the negative impact of heat stress was much higher than drought stress for most of the physiological traits (Figures 4A–D). A significant reduction in critical physiological parameters, i.e., net photosynthetic rate, stomatal conductance, and transpirational rate, was observed under stress conditions compared to control (Figures 4A–D). However, the drastic effects of drought stress were more severe than the individual effects of heat stress. However, the adverse effects of drought + heat stress were more drastic than the individual drought stress. In comparison to control, drought stress alone or in combination with heat stress significantly reduced net photosynthetic rate, stomatal conductance, and transpirational rate in three maize hybrids, i.e., YH-1898, YH-5404, and P-1543, which shows their susceptibility toward drought and heat stresses (Figures 4A–D).

On the other hand, YH-5427 and YH-5482 showed promising performance under the same conditions. The reduction in water use efficiency (WUE) of maize hybrids was much more severe under stress conditions in comparison to control. The negative impact was significant between control and stress treatments (Figures 4A–D). Although the differential response of individual stresses and their combined effects was non-significant, the overall impact of individual drought stress was higher than the heat stress alone or in combination with drought stress. The response of two maize hybrids, YH-1898 and YH-5404, was different from other hybrids under heat stress and showed comparably more reduction in their water use efficiency than drought stress alone or combination with heat stress, depicting the susceptibility of these hybrids under drought stress (Figures 4A–D).

**FIGURE 5:**
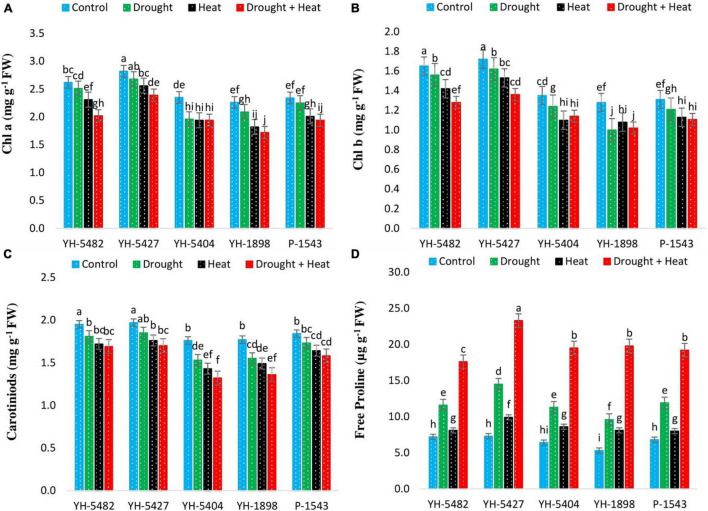
Effect of heat, drought, and heat +drought stress on **(A)** Chlorophyll an **(B)** Chlorophyll a **(C)** Carotenoid Contents, and **(D)** Free Proline Contents in five maize hybrids. Capped bars above means represent the ± SE of three replicates.

The concentration of leaf chlorophyll contents (Chlorophyll a and b) was also observed to be reduced under stress conditions compared to control (Figures 5A–D). However, the negative impact caused by the heat stress alone or in combination with drought stress was more critical than individual drought stress; still, the reduction in chlorophyll a and chlorophyll b was significant with control under combined drought + heat stress only. A similar trend in maize hybrids was also observed for Chlorophyll a, b as in Pn, Ci, and Tr, respectively, where YH-1898, YH-5404, and P-1543 showed a significant reduction in chlorophyll concentrations under stress conditions (Figures 5A–D). Leaf carotenoid contents, another important pigment, showed a reduction in their value under stress conditions. However, the difference between individual drought and heat stresses was non-significant, although heat stress proved more dangerous to carotenoid contents (Table 2). Significant reductions in carotenoid contents were only observed in YH-1898 and YH-5404 under combined drought + heat stress (Figures 5A–D).

Correlation coefficient analysis showed a strong positive correlation between grain yield and physiological parameters, i.e., net photosynthetic rate (Pn), stomatal conductance (Ci), transpirational rate (Tr), water use efficiency (WUE), chlorophyll a and b, and carotenoid contents under control as well as stress conditions (Figures 3A–D). However, a negative correlation between water use efficiency and grain yield was observed under individual drought stress and in combination with heat stress.

### Accumulation of Proline

Abiotic stresses regulate osmolytes’ accumulation, especially proline contents in maize hybrids (Figures 5A–D). Proline contents were observed to increase under drought, heat, and combined drought + heat stress in all maize hybrids. However, the increase in proline contents was more prominent under combined drought + heat stress, followed by individual drought and heat stresses, respectively. Free proline content percentage was observed to have a positive correlation with grain yield under all stress treatments. However, the degree of correlation was weaker than the control treatment (Figures 3A–D). Maize hybrid YH-5427 showed a comparatively higher accumulation of proline contents, depicting that it is more tolerant and productive than other studied hybrids.

### Accumulation of Reactive Oxygen Species and Antioxidant Activities

**FIGURE 6:**
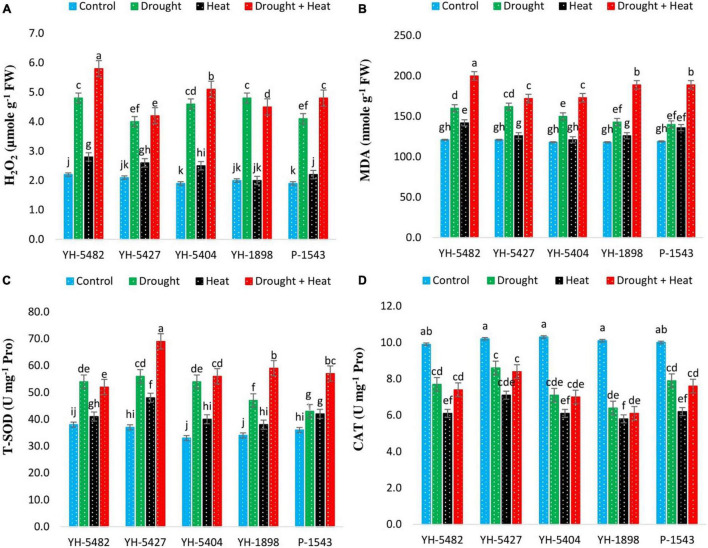
Effect of heat, drought, and heat +drought stress on **(A)** H_2_O_2_ **(B)** MDA **(C)** T-SOD and **(D)** CAT in five maize hybrids. Capped bars above means represent the ± SE of three replicates.

The accumulation of reactive oxygen species (ROS) and their impact on degrading the cell membranes were increased significantly under stress conditions (Figures 6A–D). Although all three stress treatments (drought, heat, drought + heat) increased the induction rate of ROS accumulation in maize hybrids, however, interactive drought + heat stress posed a more serious threat of ROS accumulation than individual stresses. At individual stress levels, drought stress was more critical than heat stress as H_2_O_2_, and MDA accumulation was higher in maize hybrids under drought stress than heat stress (Figures 6A–D). More significant oxidative damage due to H_2_O_2_ and MDA in YH-5482, YH-5404, and P-1543 indicates their susceptibility to the individual as well as combined heat and drought stresses.

Higher antioxidant activity was observed in maize hybrids under all stress conditions. Total superoxide dismutase (T-SOD) was significantly increased under stress conditions compared to control (Figures 6A–D). The highest activity of T-SOD was recorded in combined drought + heat stress conditions compared to individual drought and heat stresses. Maize hybrid YH-5427 showed maximum activity of T-SOD followed by YH-5404 and YH-5482, while P-1543 and YH-1898 showed a minimum overall increase in T-SOD. However, Catalase (CAT) activity was significantly reduced in all maize hybrids under all stress conditions compared to control (Figures 6A–D). The highest reduction in catalysis activity was observed under heat stress compared to drought stress and combined drought + heat stress. Maize hybrid YH-5427 showed a minimum reduction in catalysis activity while YH-1898 showed the maximum reduction. The correlation graphs unveiled the negative relationship of ROS species, i.e., H_2_O_2_ and MDA, with grain yield under all stress conditions. At the same time, total superoxide dismutase (T-SOD) and Catalysis (CAT) showed a significantly positive correlation with grain yield under stress conditions (Figures 3A–D). However, the degree of association might vary a bit depending upon the type of stress.

### Kernel Quality Traits

**FIGURE 7:**
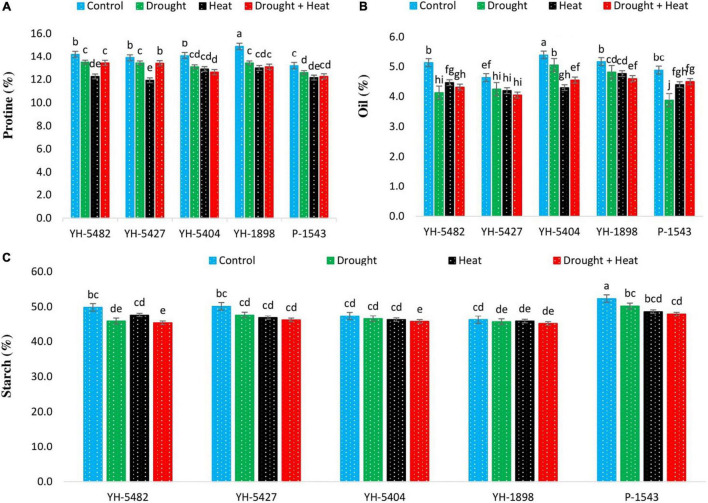
Effect of heat, drought, and heat +drought stress on **(A)** Protein contents % **(B)** Oil contents % and **(C)** Starch contents % in five maize hybrids. Capped bars above means represent the ±SE of three replicates.

Compared with control, drought, heat, and combined drought + heat stresses were observed to have reduced kernel quality traits (kernel protein content percentage, kernel oil content percentage, and kernel starch content percentage) (Figures 7A–C). However, the response of kernel quality traits to each stress was different. A significant reduction in kernel protein content percentage was observed in YH-5427 and YH-5482 under heat stress conditions. Although heat stress was also one of the factors in reducing the protein % in other maize hybrids, their impact, however, was non-significant with individual drought and combined drought + heat stresses (Figures 3A–D). For kernel oil content percentage, the response of individual and combined stresses was quite variable. Compared to control, kernel oil content percentage was reduced in all three stress conditions, but the effect was more evident in YH-5482, YH-5404, and P-1543, showing low stability of kernel oil content percentage under stress conditions. Compared to control, a non-significant reduction in maize hybrids was reported in kernel starch content percentage under all three stress conditions except for P-1543, where differences among control and stress treatments were significant (Figures 7A–C). However, combined drought + heat stress showed more critical than individual stresses. Maize hybrid P-1543 proved to be more sensitive to abiotic stresses for kernel quality traits (Figures 7A–C).

## Discussion

Sustainable crop production under the changing climatic conditions is one of the key points to feed the immensely growing world population. The unpredictability of climatic events like heat waves, drought, and floods make it challenging for plant scientists to develop a multifactor tolerant variety. Among the environmental factors, drought and heat stress alone or in combination, are the key factors limiting crop productivity and sustainability. Although drought and heat stresses affect the overall growth and development of maize crop, including maize, the reproductive stage is more severely affected by these stresses (Barnabás et al., 2008). Several researchers suggested a comprehensive study of heat, drought and combined drought + heat stress to get detailed, in-depth knowledge of these stresses and their effects on the individual as well as interactive levels (Dreesen et al., 2012). In the current study, individual drought and heat stresses and their combination were observed to have a severely negative impact on plant height, ear height, ear length, number of kernels per row, number of rows per ear, number of kernels per ear, thousand kernel weight, and eventually, kernel yield per hectare in five maize hybrids, however, the adverse effects of combined drought + heat stress were more damaging than their individual effects (Table 2). Results revealed that the adverse effects of stress treatments were more critical for P-1543 and YH-1898, while YH-5427 showed promising performance under these stress treatments. Previously, several researchers reported a significant reduction in maize kernel yield and related attributes under drought and heat stress conditions at the flowering/anthesis stage (Sah et al., 2020; Yousaf et al., 2021). Furthermore, several other studies showed highly adverse effects of combined drought + heat stress on growth and yield-related traits in maize (Meseka et al., 2018; Hussain et al., 2019; Nelimor et al., 2020). However, the extent of damage caused by these stresses depends on the severity of stress, stage exposure, and hybrids/verities under study.

In the current study, a severe reduction in leaf pigments, i.e., Chl a, Chl b, and carotenoid contents, were observed under individual drought and heat stress as well as combined drought + heat stress conditions. However, the impact of concurrent drought + heat stress was also much more radical than their individual stresses. Chlorophyll is one of the most essential plant pigments required for photosynthesis in plants, which is one of the most heat-sensitive physiological processes. High temperature decreases the chlorophyll contents, reduces light quanta’s interception, decreases the net photosynthetic rate, and ultimately results in lower kernel yield (Hasanuzzaman et al., 2013). Drought stress also reduces the chlorophyll contents, but its degree of reduction is lower than heat stress (Lamaoui et al., 2018).

Results revealed the differential response of physiological processes, including net photosynthetic rate, transpirational rate, stomatal conductance, and water use efficiency under stress treatments (Figures 4A–D). It was observed that all three stress treatments (drought, heat, drought + heat) negatively affected the performance of physiological processes but the impact of combined drought + heat stress was much more severer. Transpirational rate and stomatal conductance were found higher than control under drought stress, indicating the regulation of the stomatal opening and rapid movement of water from roots to air through the maize plant (Yang et al., 2012). On the other hand, water use efficiency was significantly reduced under drought stress conditions, revealing that maize plants are much more sensitive to drought stress than individual heat or combined drought + heat stresses, as investigated by Lawas et al. (2018). Considering the impact of stress treatments on the physiological functioning of maize hybrids, YH-5427 was proved to be the most promising hybrid as it showed considerably higher performance under all three stress treatments while P-1543 showed poor performance followed by YH-5404. Ghani et al. (2020) also unveiled the heat tolerance ability of maize hybrid YH-5427.

Proline, an essential amino acid, is one of the most widely used indicators of drought stress and irrigation scheduling. It accumulates more rapidly than other amino acids under water-deficient conditions and acted as an osmoregulator under abiotic stress conditions (Meena et al., 2019). In the current study, a considerably higher free proline accumulation was observed in all maize hybrids under stress conditions than in control. However, the highest accumulation was recorded in combined drought + heat conditions followed by individual drought stress (Figures 5A–D). Moreover, local maize hybrid YH-5427 accumulated more proline than the other hybrids, indicating its ability to accumulate stress osmolytes to achieve higher stress tolerance as reported by Chun et al. (2018).

Abiotic stresses, especially drought and heat stress, could lead to overproduction of ROS, which will have a disastrous effect on the physiological functioning of plants, and crop productivity might get severely affected. The balance between ROS production and antioxidant enzyme activity is crucial to keep plants functioning normally under stressed conditions because an increased ROS production rate than antioxidant enzyme activity could seriously damage several cellular components (Das and Roychoudhury, 2014). Plants use complex antioxidant defense mechanisms to check the unrestricted production of ROS to defend plants from severe oxidative stress (Sharma et al., 2012). Increased production of MDA and H_2_O_2_ was observed under all stressed conditions compared to control, which indicates oxidative stress in maize hybrids. However, MDA and H_2_O_2_ production were higher in combined drought + heat stress than individual drought and heat stresses. The higher MDA levels in plants indicate increased lipid peroxidation under stress conditions (Thussagunpanit et al., 2015). This happens due to the unfavorable variations in climatic conditions, which lead to the overproduction of ROS and membranes instability due to the polarization of lipid bilayers. In recent few years, H_2_O_2_ gained considerable attention due to the small size of its molecules and the highest half-life (1 ms) among reactive oxygen species (ROS). H_2_O_2_ is produced via superoxide and superoxide dismutase, type III peroxidases, or directly released by some oxidases. Although H_2_O_2_ is a regulator of many physiological plant functions, i.e., strengthening the cell wall, photosynthesis, stomatal regulation, and cell cycle, its overproduction could trigger chloroplast, peroxisome autophagy, and programmed cells death (Das and Roychoudhury, 2014). However, H_2_O_2_ could be removed by catalase, peroxiredoxin, glutathione peroxidase-like enzymes, and ascorbate peroxidase.

In the current study, kernel protein, oil, and starch content percentage were observed to be reduced under all stress treatments, either individual or combined (Figures 7A–C). Although the difference between different stress treatments was non-significant, heat stress alone and in combination with drought stress proved more deleterious. It might be due to reduced grain weight, reduced carbohydrate reserves in the stem, alteration in grain composition, and increased water loss rates from kernels, resulting in the inhibition of ADP glucose pyrophosphorylase and soluble starch synthase activity, thus disturbing the source-sink relationship (Barnabás et al., 2008; Sehgal et al., 2018). Furthermore, the amount of starch and protein accumulation in grains, which depends on the total number of endosperm cells and the final size of these cells, is highly affected by abiotic stresses especially heat stress (Farooq et al., 2017). Heat stress also limits the production of storage cells by restricting the accumulation of grain reserves (Sehgal et al., 2018).

Correlation coefficient graphs explain the association of kernel yield and associated traits under heat, drought, and heat + drought stress conditions (Figures 3A–D). Few plant traits, i.e., the number of kernels per ear, net photosynthetic rate, transpirational rate, stomatal conductance, chlorophyll a & b, carotenoid contents, T-SOD and CAT showed a significantly high and positive correlation with grain yield under all stress conditions. This higher correlation might be due to higher stability of leaf pigments, i.e., chlorophyll a & b, carotenoid contents under stress conditions help the plant stabilize photosynthetic ability through stomatal regulation and adjusted transpirational rate, which ultimately increases the number of kernels per ear and grain yield per plant. Similar findings were also reported by Ashraf and Harris (2013), and Maghsoudi et al. (2016). Moreover, higher antioxidant activity, i.e., total superoxide dismutase and catalase, helped the plants to overcome the negative effects of oxidative stress caused by ROS (Das and Roychoudhury, 2014).

## Conclusion

In conclusion, the impact of exposure of maize hybrids to drought, heat, and drought + heat stress at pre-flowering was very lethal, impairing plant growth and development by affecting kernel yield and its contributing traits, i.e., photosynthetic ability, accumulation of compatible osmolytes, enzymatic antioxidant activity and kernel quality traits. Among the individual stresses, the effects of heat stress on maize plants were more severe than drought stress. However, the concurrent effects of drought and heat stress on morpho-physiological, phenological and biochemical, traits were far more severe than their individual effects. Interestingly, the effects of individual and combined stresses on kernel quality traits were not as severe as in other traits. The comparative performance of local maize hybrid YH-5427 was much higher than other hybrids under drought and/or heat stress conditions attributing to its higher photosynthetic pigments’ stability and efficiency and strong antioxidant and osmolytes accumulation-based defense mechanisms. Therefore, it is recommended to cultivate the heat and drought-tolerant hybrids, i.e., YH-5427 and YH-5482 in heat and drought-prone areas of the country.

## Data Availability Statement

The original contributions presented in the study are included in the article/supplementary material, further inquiries can be directed to the corresponding authors.

## Author Contributions

MIY, MWR, and YJ initiated and designed the research, and were substantial contributors to the preparation of the manuscript. MIY, AS, and GR performed the experiment, analyzed the data, and wrote the manuscript. MZA, SH, and SAS provided advice on the experiments. MAM and MY did formal data analysis. IA contributed to the acquisition of the data and participated in the revision of the manuscript. All authors contributed to the article and approved the submitted version.

## Conflict of Interest

The authors declare that the research was conducted in the absence of any commercial or financial relationships that could be construed as a potential conflict of interest.

## Publisher’s Note

All claims expressed in this article are solely those of the authors and do not necessarily represent those of their affiliated organizations, or those of the publisher, the editors and the reviewers. Any product that may be evaluated in this article, or claim that may be made by its manufacturer, is not guaranteed or endorsed by the publisher.
